# Up-regulation of the Hippo pathway effector TAZ renders lung adenocarcinoma cells harboring EGFR-T790M mutation resistant to gefitinib

**DOI:** 10.1186/2045-3701-5-7

**Published:** 2015-02-05

**Authors:** Wei Xu, Yunyan Wei, Shuangshuang Wu, Yun Wang, Zhen Wang, Yu Sun, Steven Y Cheng, Jianqing Wu

**Affiliations:** Department of Geriatrics, The First Affiliated Hospital of Nanjing Medical University, 300 Guangzhou Road, Nanjing, Jiangsu 210029 China; Department of Developmental Genetics, School of Basic Medical Sciences, Nanjing Medical University, 140 Hanzhong Road, Nanjing, Jiangsu 210029 China; Department of Pathology, The First Affiliated Hospital of Nanjing Medical University, 300 Guangzhou Road, Nanjing, Jiangsu 210029 China; Origin Biosciences Inc., 5 New Model Road, Nanjing, Jiangsu 210009 China

**Keywords:** TAZ, EGFR, EMT, Gefitinib, Lung adenocarcinoma

## Abstract

**Background:**

The T790M mutation of epithelial growth factor receptor (EGFR) is a major cause of the acquired resistance to EGFR tyrosine kinase inhibitor (EGFR-TKIs) treatment for lung cancer patients. The Hippo pathway effector, TAZ, has emerged as a key player in organ growth and tumorigenesis, including lung cancer.

**Results:**

In this study, we have discovered high TAZ expression in non-small cell lung cancer (NSCLC) cells harboring dual mutation and TAZ depletion sensitized their response to EGFR-TKIs. Mechanistically, knockdown of TAZ in T790M-induced resistant cells leaded to reduced anchorage-independent growth *in vitro*, tumor formation and resistance to gefitinib *in vivo*, correlated with epithelial-mesenchymal transition (EMT) and suppressed migration and invasion. Furthermore, we confirmed CTGF and AXL, novel EMT markers and potential therapeutic targets for overcoming EGFR inhibitor resistance, as directly transcriptional targets of TAZ.

**Conclusions:**

Taken together, this study suggests that expression of TAZ is an intrinsic mechanism of T790M-induced resistance in response to EGFR-TKIs. Combinational targeting on both EGFR and TAZ may enhance the efficacy of EGFR-TKIs in acquired resistance of NSCLC.

**Electronic supplementary material:**

The online version of this article (doi:10.1186/2045-3701-5-7) contains supplementary material, which is available to authorized users.

## Background

Non-small cell lung cancer (NSCLC) is the leading cause of cancer-related deaths worldwide. Despite the benefits shown with epidermal growth factor receptor tyrosine kinase inhibitor (EGFR-TKI) treatment in patients with TKI-sensitive EGFR mutations such as delE746-A750 (exon 19) and L858R (exon 21), most patients ultimately relapse due to the development of drug resistance. A secondary point mutation in exon 20 of EGFR, which leads to substitution of methionine for threonine at position 790 (T790M), accounted for more than half of the cases with NSCLC who developed acquired resistance to the first-generation EGFR-TKIs (gefitinib and erlotinib) [1]. The second-generation EGFR-TKIs, which form covalent, irreversible bonds with the target, are thought to be one strategy to overcome resistance. However, mounting studies have shown their limited activity in cells with T790M mutations [2] given acquiring ligand-independent oncogenic potential [3], more aggressive tumor phenotype and affecting other pathways [4]. Therefore, to develop an effective therapy for patients harboring EGFR T790M is important to overcome the acquired resistance.

The transcriptional co-activator with PDZ-binding motif (TAZ), also known as WWTR1 for WW domain containing transcription regulator 1, was originally identified as a 14-3-3-binding proteins. Both TAZ and its paralog, Yes-associated protein (YAP), are key effectors of the Hippo pathway. Unlike YAP, knock-out of TAZ gene in mice does not overtly affect mouse development or fertility but selectively affects the construction and function of the lung and kidney [5]. TAZ is known to bind to a variety of transcription factors to modulate mesenchymal stem cell differentiation [6], self-renewal of embryonic stem cell [7], crosstalk with other signaling pathways [8] and mechanotransduction [9].

Recent studies showed that TAZ promoted lung cancer/epithelial cell proliferation *in vitro* and tumor development *in vivo*[10]. Higher TAZ expression was associated with poor differentiation, shorter survival and metastasis in lung cancer patients [11]. Lately, microarray screening demonstrated that EGFR ligands (amphiregulin, epiregulin and neuregulin 1) were downstream targets of TAZ and EGFR signaling pathway was activated in NSCLC tissues with high TAZ expression [12, 13]. However, the roles of TAZ in therapeutic response of EGFR-TKI have not been explored. In this study, we provide evidence that TAZ-mediated tumorigenesis and progression correlated with gefitinib sensitivity in lung adenocarcinoma cells harboring EGFR T790M mutation.

## Results

### High TAZ expression in NSCLC cells harboring EGFR T790M mutation

**Figure 1:**
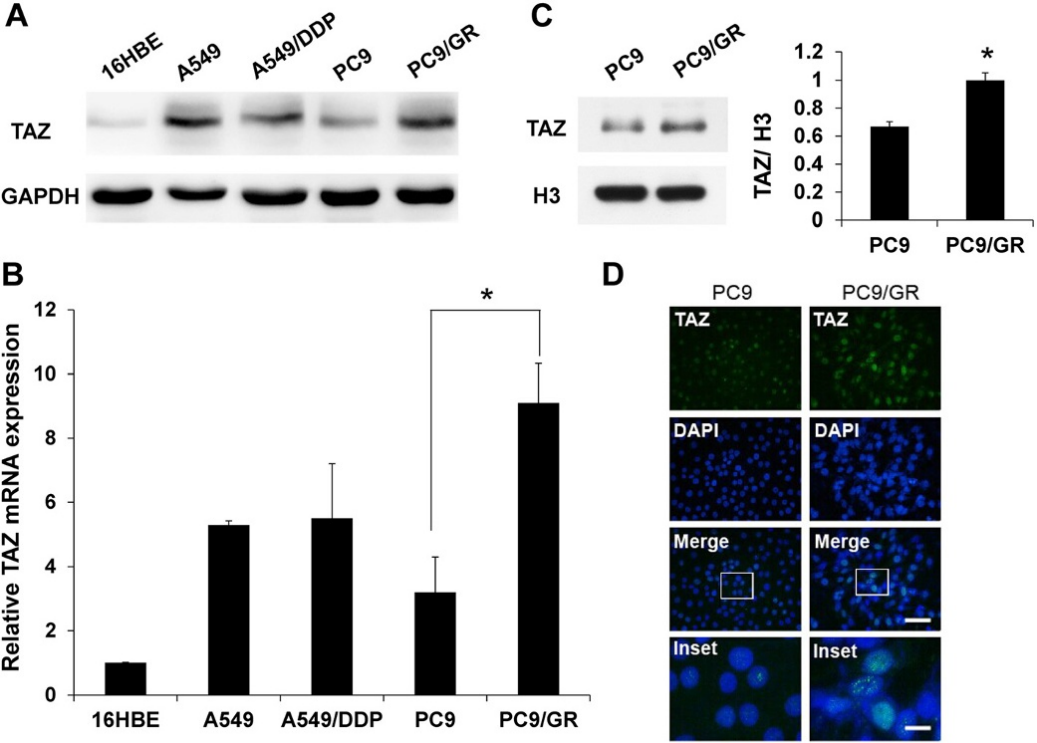
**Enhanced expression of TAZ in gefitinib-resistant cell. (A)** Lysates derived from human bronchial epithelial cell line 16HBE, lung adenocarcinoma cell lines A549, cisplatin-resistant A549/DDP, gefitinib-sensitive PC9 and gefitinib-resistant PC9/GR were analyzed by western blot using anti-TAZ antibodies. The levels of GAPDH were detected as loading controls. **(B)** Relative mRNAs of TAZ in lung cancer cell lines were examined by real-time PCR. The endogenous b-actin RNA was used as the internal control. **(C)** Nuclear fractions in PC9 and PC9/GR cell lines were analyzed by western blot. The levels of histone H3 were detected as loading controls. Densitometric evaluation of TAZ:H3 ratios is illustrated in graph beside. Data are shown as means ± SEM. n=3. Statistical analyses were carried out using Student’s t-test. Significance: * P<0.05. **(D)** Confocal microscopy of immunofluorescent staining of TAZ in PC9 and PC9/GR cells. Scale bar = 100 mm for original picture and 25 mm for inset. Dapi was used to stain nuclei.

To explore the expression of TAZ in gefitinib-sensitive and resistant NSCLC, we first examined the endogenous expression of TAZ by western blot (Figure 1A) and real-time PCR (Figure 1B) using several lung cell lines, including human bronchial epithelial cell line 16HBE, lung adenocarcinoma cell lines A549, cisplatin-resistant A549/DDP, gefitinib-sensitive PC9 (EGFR delE746-A750) and gefitinib-resistant PC9/GR derived from PC9 harboring T790M mutation (Additional file 1: Table S1). We found that TAZ expressed in all NSCLC cell lines, especially relative level of TAZ mRNA in PC9/GR was 2.84-fold higher than that in PC9 cells. The functional role of TAZ is different according to cellular localization. We subsequently compared the nuclear accumulation of TAZ between PC9 and PC9/GR by immunoblot analysis using nuclear fractions (Figure 1C). Gray value analysis suggested TAZ nuclear protein level in PC9/GR was 1.50-fold higher than that in PC9. Confocal microscopy also showed that the majority of the TAZ protein in PC9/GR is localized in the nucleus using immunofluorescence staining (Figure 1D).

### Effect of TAZ on sensitivity of NSCLC cells to gefitinib

**Figure 2:**
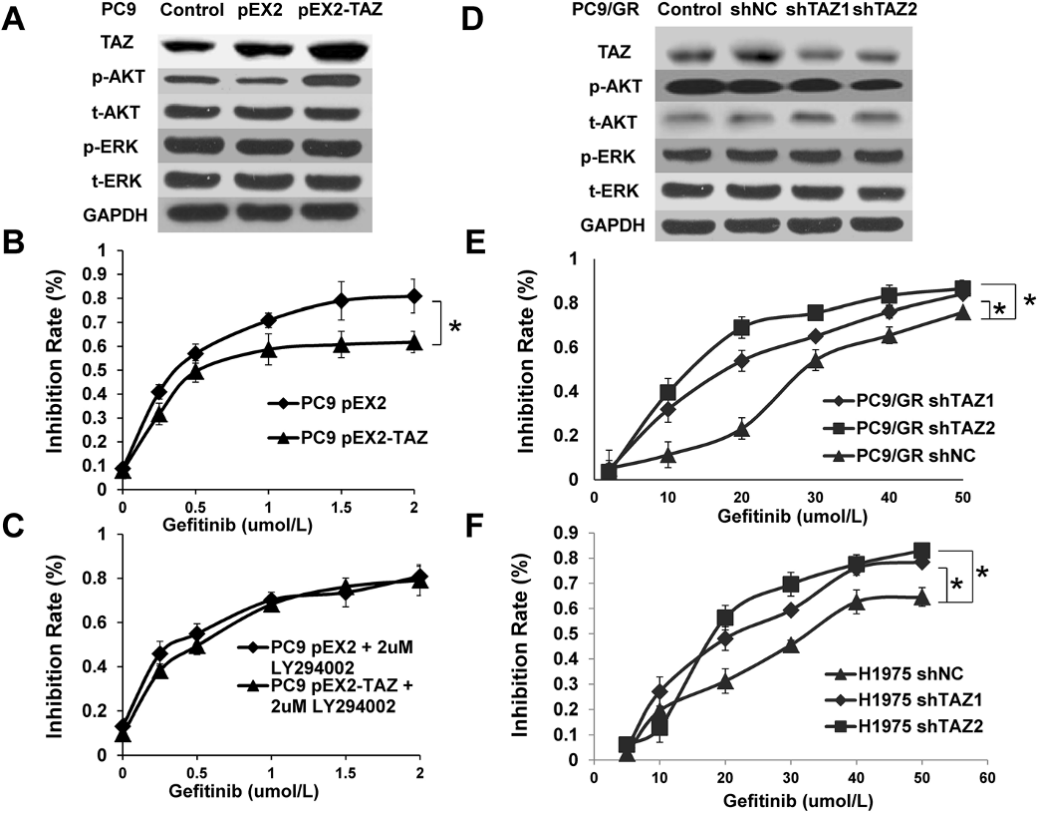
**Effect of TAZ on the sensitivity of NSCLC cells to gefitinib. (A)** Protein lysate extracted from PC9 cells expressing pEX2-TAZ were subjected to western blot by using anti-TAZ, anti-pAkt, anti-tAkt, anti-pERK and anti-tERK antibodies, respectively. **(B)** Overexpression of TAZ descreased gefitinib sensitivity in PC9 cells. Cells were transfected and then exposed to various doses of gefitinib for 48 h and viability was accessed by MTT assay as described in Methods. The inhibition rate was calculated according to the following formula: inhibition ratio (%) = [1- A490 (experimental group)/A490 (Control group)] × 100. **(C)** LY294002, as a inhibition of Akt signaling, abolished TAZ-driven descreased sensitivity to gefitinib of PC9 cell. **(D)** shRNAs against different regions of TAZ mRNA (shTAZ1 and shTAZ2) were expressed in PC9/GR cells. Expressions of TAZ, pAkt, tAkt, pERK and tERK were detected by western blot. **(E)** enhanced sensitivity of PC9/GR cells to gefitinib after TAZ knockdown. **(F)** Enhanced sensitivity to gefitinib after TAZ knockdown were detected in H1975 cells. Procedures and conditions for inhibition rate are described as in **(B)**. Results are expressed as means ± SEM from three independent experiments (*P<0.05). Statistical analyses were carried out using Student’s t-test.

To confirm that TAZ was responsible for gefitinib sensitivity in lung cancer cells, we first overexpressed TAZ in PC9 cells and knocked down TAZ in TAZ-high/drug-resistant PC9/GR, detecting effects by western blot. Meanwhile, immunoblot for pAkt, tAkt, pERK and tERK, downstream intracellular signaling pathways of EGFR, showed that pAkt level correlated with expression of TAZ (Figure 2A and D). Compared with control (PC9 pEX2), overexpression of TAZ in PC9 (PC9 pEX2-TAZ) reduced sensitivity to growth inhibition induced by gefitinib (Figure 2B). The half maximal inhibitory concentration (IC50) values of PC9 cells to gefitinb in control and pEX2-TAZ group were 0.32 ± 0.01 μmol/L and 0.57 ± 0.01 μmol/L, respectively. LY294002, as a inhibition of Akt signaling, abolished descreased sensitivity to gefitinib of TAZ-driven in PC9 cell (Figure 2C). Knockdown of TAZ by both shTAZ1 and shTAZ2 in PC9/GR cells sensitized their response to gefitinib (Figure 2E). The IC50 values of PC9/GR cells to gefitinib in negative control (shNC), shTAZ1 and shTAZ2 group were 29.18 ± 0.08 μmol/L, 18.26 ± 0.09 μmol/L and 12.19 ± 0.18 μmol/L, respectively. Meanwhile, knockdown of TAZ in H1975 cells harboring EGFR-T790M also sensitized their response to gefitinib (Figure 2F).

### TAZ is important for tumorigenesis and resistant to gefitinib of PC9/GR

**Figure 3:**
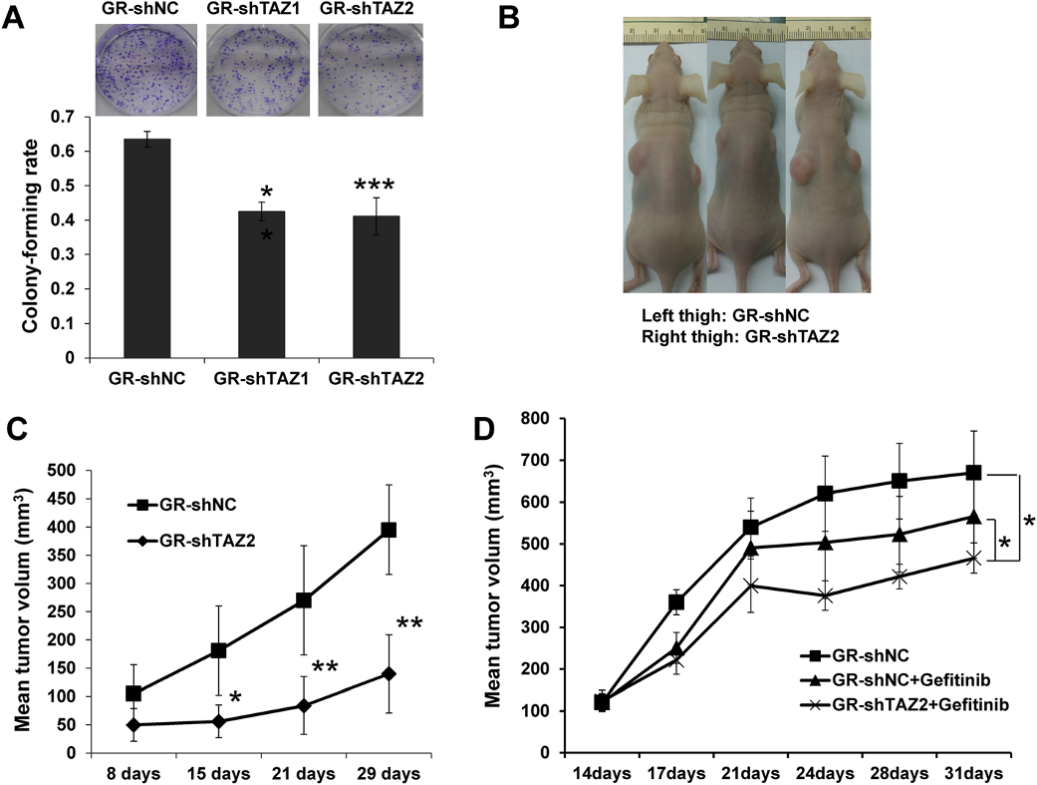
**TAZ knockdown in PC9/GR cells suppresses anchorage-independent growth, tumorigenesis and resistant to gefitinib in vivo. (A)** Two hundred stable GR-shNC, GR-shTAZ1 and GR-shTAZ2 cells were plated onto 6-well plates and a colony formation assay was assessed and photographed. The rate of colony formation was calculated as follows: colony-forming rate = (average colony number/ plated single cell number) × 100%. The colony-forming rates of GR-shTAZ cells were reduced drastically compared with control group. Results are expressed as means ± SEM from three independent experiments (**P<0.01, ***P<0.001). **(B)** PC9/GR cells with stable expression of shTAZ2 and shNC were inoculated subcutaneously into right and left flanks of nude mice (n = 6 per group), respectively. Representative photographs of nude mice 29 days after inoculation are shown. **(C)** Tumor growth curve is indicated. **(D)** Mice bearing PC9/GR xenografts were treated daily with gefitinib at indicated doses. Data represent means ± SEM (*P<0.05, **P<0.01). Statistical analyses were carried out using Student’s t-test.

To examine the importance of TAZ in the tumorigenesis of PC9/GR cells, we generated stable cells (PC9/GR-shNC, PC9/GR-shTAZ1 and PC9/GR-shTAZ2) by G418 selection. We assessed the anchorage-independent growth capability of PC9/GR-shTAZ cells. PC9/GR-shNC cells grew well in soft agar, whereas colony-forming rates were reduced dramatically for PC9/GR-shTAZ1 and PC9/GR-shTAZ2 compared with control group (P<0.01 and P<0.001, respectively) (Figure 3A). We subsequently examined whether TAZ contributes to tumor formation in nude mice. PC9/GR-shTAZ2 cells and PC9/GR-shNC cells were separately injected into the thigh of nude mice and the growth of the tumors was monitored. Compared with PC9/GR-shNC group (right thigh), PC9/GR-shTAZ2 group (left thigh) were compromised in forming tumors at 29th days following the injection (tumor volume: 301.31 ± 46.00 mm^3^ versus 78.13 ± 20.23 mm^3^, Figure 3B). The mean tumor growth curve of the resulting tumors was shown in Figure 3C. Two groups of tumor volume showed statistically significant difference in terms of survival. To test the resistant changes in vivo, mice bearing established PC9/GR-shNC and PC9/GR-shTAZ2 tumor xenografts were treated orally with gefitinib. Response to treatment was assessed by tumor growth inhibition. These results support the suggestion that down-regulation of TAZ sensitize gefitinib in vivo (Figure 3D).

### TAZ mediates epithelial-mesenchymal transition (EMT) of gefitinib-resistant PC9/GR

**Figure 4:**
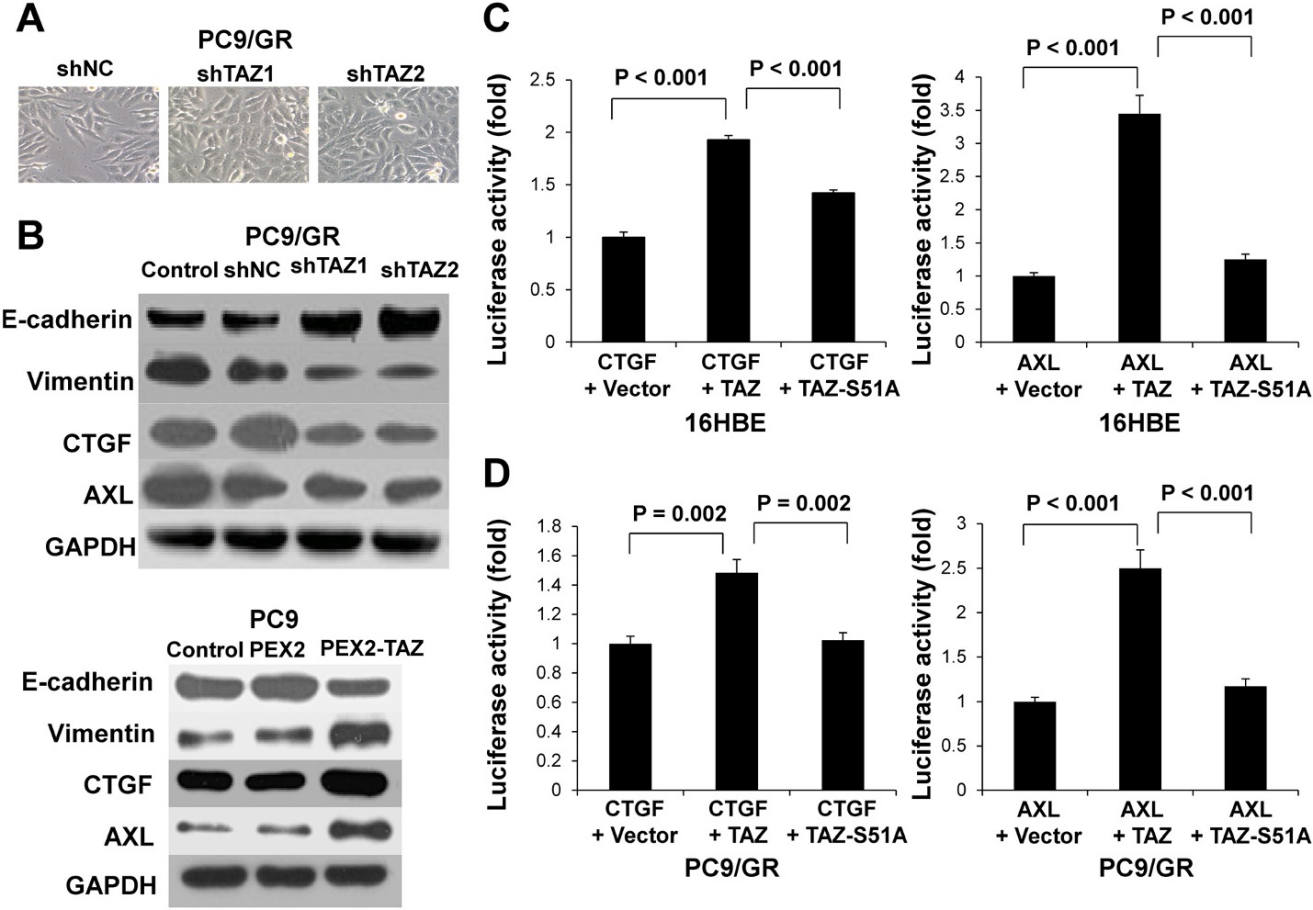
**TAZ mediates EMT of gefitinib-resistant PC9/GR. (A)** TAZ knockdown in PC9/GR cells resulted in a morphology change in PC9/GR by a light microscope. The spindle form of the PC9/GR-shNC cells changed to a round shape. The cell density of the clusters was obviously enhanced in the PC9/GR-shTAZ cells. **(B)** Cell lysates from PC9/GR and PC9 cells expressing vector, shTAZ and PEX2-TAZ were separated and probed with antibodies for epithelial marker and mesenchymal markers as indicated. TAZ knockdown in PC9/GR cells increased E-cadherin and decreased vimentin, CTGF and AXL. All data were normalized to GAPDH. **(C)** Luciferase reporter assay in 16HBE cells with TAZ or TAZ-S51A overexpression. 16HBE cells co-transfected with pGL3-CTGF/pGL3-AXL and pEX2-TAZ showed significantly elevated reporter activity compared with controls. Meanwhile, cells co-transfected with pEX2-TAZ-S51A, which disrupts the TEAD binding, exhibited decreased luciferase activity compared with pEX2-TAZ. Cells were harvested 48 h after transfection for measurement of luciferase activity in all experiments. **(D)** Luciferase reporter assay in PC9/GR cells. Total amounts of DNA and RNA were kept constant. Results are expressed as means ± SEM from three independent experiments. Statistical analyses were carried out using Student’s t-test.

Since expression of TAZ promoted EMT in breast cancer cells and was responsible for their resistance to Taxol, we analyzed these changes induced by TAZ knockdown in PC9/GR cells. We observed morphologic differences between the control and TAZ knockdown cells using a light microscope. The spindle form of the PC9/GR-shNC cells changed to a round shape and TAZ knockdown in PC9/GR cells resulted in clusters of more densely compact sheets of cells, suggesting that epithelioid-like morphological changes might have occurred (Figure 4A). We subsequently analyzed the expression of epithelial and mesenchymal marker proteins using western blot. Compared with the PC9/GR-shNC cells, E-cadherin expression was drastically increased and vimentin expression was reduced in TAZ knockdown cells. In addition, two factors, connective tissue growth factor (CTGF) and anexelekto (AXL), which were considered to be the target genes of TAZ and induce EMT, were decreased by western blot (Figure 4B) and real-time PCR. We also detected these proteins in PC9 cells with overexpression of TAZ (Figure 4B). To further demonstrate transcriptional activity of TAZ on CTGF and AXL expression, we conducted luciferase reporter assays in 16HBE (Figure 4C). The pGL3-basic Luc vector subcloned with the CTGF/AXL promoter and the pEX2-TAZ or pEX2-TAZ-S51A plasmids were co-transfected into each cell line, and after 48 h we measured the luciferase activities. The luciferase activities of the CTGF/AXL promoter were significantly enhanced in 16HBE when co-transfected with the TAZ gene, but not with the vector control and pEX2-TAZ-S51A, which disrupts the TEAD transcription factors binding (P < 0.001, respectively). The results also were confirmed in PC9/GR cells (Figure 4D).

### Knockdown of TAZ in PC9/GR suppresses cell migration and invasion

**Figure 5:**
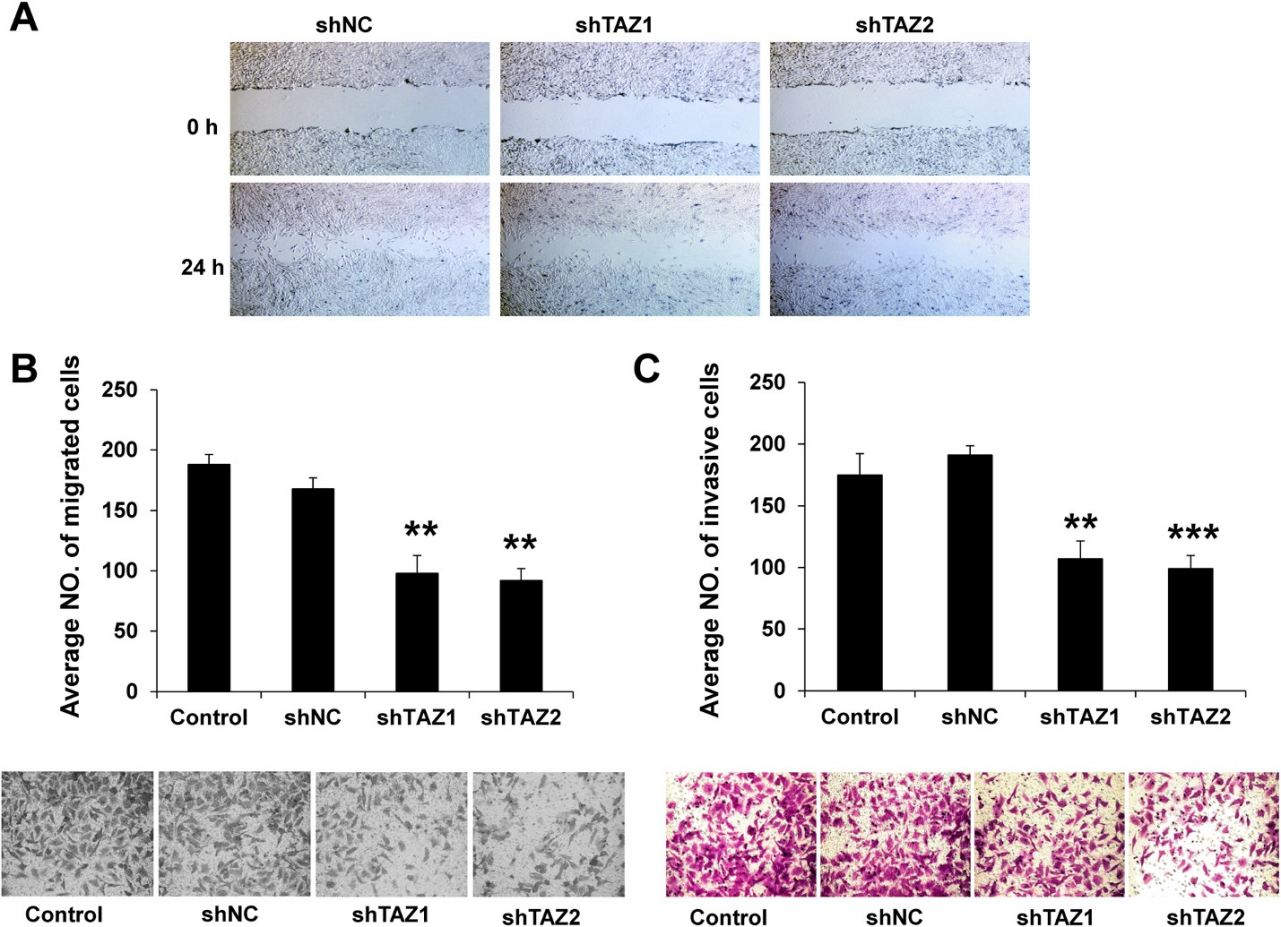
**Knockdown of TAZ in PC9/GR suppresses cell migration and invasion. (A)** Wound-healing migration assay for PC9/GR cells expressing shNC, shTAZ1 or shTAZ2. The healing of wounds by migrated cells at time 0 and 24 h was imaged. PC9/GR cells expressing shNC migrated faster than PC9/GR cells knocking down TAZ. **(B,C)** The migration **(B)** and invasion **(C)** of control, PC9/GR cells expressing shNC, shTAZ1 or shTAZ2 were assessed by transwell assay and matrigel invasion assay. The migration and invasiveness of TAZ-knocked-down PC9/GR cells decreased 52% - 63% and 53% - 62%, respectively, compared with control cells. Results are expressed as means ± SEM from three independent experiments (** P<0.01, *** P<0.001). Statistical analyses were carried out using Student’s t-test.

Next, we examined migratory and invasive potential, which are considered functional hallmarks of EMT. We compared the cell mobility using the wound healing assay (Figure 5A). The mobility of PC9/GR-shTAZ cells was dramatically decreased. Within 24 hours, the area of the wound was significantly recovered by the migrating shNC group cells. In marked contrast, the wound closure of TAZ-knocked-down PC9/GR cells was only partial within 24 hours. The motility and invasiveness of these cells were independently assessed using the Transwell migration (Figure 5B) and matrigel invasion assay (Figure 5C). Compared with control, the migration and invasiveness of TAZ-knocked-down cells decreased 52% - 63% (PC9/GR-shTAZ1 versus PC9/GR-shNC: P = 0.007, PC9/GR-shTAZ2 versus PC9/GR-shNC: P = 0.001) and 53% - 62% (PC9/GR-shTAZ1 versus PC9/GR-shNC: P = 0.003, PC9/GR-shTAZ2 versus PC9/GR-shNC: P < 0.001), respectively.

## Discussion

Although tremendous progress has been made toward understanding the molecular mechanism underlying NSCLC development and treatment, the overall survival of lung cancer patients is still significantly hindered because of the evolution of drug-resistant tumors. T790M mutation-mediated gefitinib resistance accounts for 50% of TKI resistance in patients carrying an EGFR mutation. Accumulating evidence suggests that T790M-induced resistance is not only caused by lowering the affinity for gefitinib to nullify the hypersensitivity of activating EGFR mutations [14], but also attributed to the gain of transforming activity [2], potent proliferative and progressive advantage to cells expressing the T790M double mutants, with increasing ligand-independent receptor activation [3, 4].

Recently, TAZ and its paralogue YAP have been identified as major proteins mediating the effects of the novel Hippo tumor suppressor pathway. Several studies showed that TAZ amplification existed in lung cancer, driving the progression and metastasis of lung tumor-propagating cells [15] and significantly correlated with the prognosis of patients [11]. In this report, we have for the first time identified TAZ as a novel gene responsible for sensitivity of gefitinib in NSCLC harboring T790M mutation. By overexpressing TAZ in gefitinib-sensitive PC9 cells, we found that enhanced levels of TAZ directly reduced sensitivity to growth inhibition induced by gefitinib, which abolished by the inhibition of Akt signaling. On the other hand, knockdown of endogenous TAZ in drug-resistant cells sensitized these cells to gefitinib. Therefore, targeting TAZ in TAZ-overexpressed, T790M-induced resistance might be a novel therapeutic strategy for sensitizing EGFR-TKI treatment.

Consistent with our finding, some proteins in the Hippo-LATS pathway have also been shown to mediate the drug sensitivity in human cancers. For example, Yang et al. have reported that TAZ and its downstream transcriptional targets cysteine-rich 61 (Cyr61) and CTGF mediated Taxol resistance in breast cancer cells [16]. In a screen of shRNAs, LATS1, a negative regulator of TAZ, was identified as one of the genes causing paclitaxel resistance upon knockdown in A549 cells [17]. Similarly, overexpression of YAP, the TAZ paralogue, has been found to involve in drug response of mammary [18], ovarian [19, 20], colon [21] and hepatocellular [22–25] cancers to multiple chemotherapeutic and targeted therapies. In addition, loss of other important tumor suppressor genes in the Hippo pathway such as Mst1, hEx and RASSF1A leads to drug resistance, whereas overexpression of CD44 or Itch, the negative regulators of the Hippo pathway, induces drug resistance [26]. Together, these studies suggest that the emerging Hippo pathway may have significant roles in drug sensitivity of human cancers. Therefore, it will be very interesting to examine how TAZ is responsible for gefitinib sensitivity in NSCLC.

In current study, we provided evidence that endogenous TAZ contributed to the tumorigenicity of NSCLC cells harboring T790M mutant, as specific knockdown of TAZ leaded to reduced anchorage-independent growth *in vitro* and tumor formation *in vivo*. Preclinical and clinical data suggest that markers of EMT may be associated with limited responses to EGFR-TKI, whereas retention of an epithelial phenotype is associated with response even in patients without EGFR receptor mutations [27]. Accumulating evidence suggests TAZ promotes EMT-mediated cancer progression [28, 29]. Consistent with these conclusion, our data showed knockdown of TAZ in PC9/GR was correlated with EMT as the transfectants demonstrated enhanced expression of epithelial marker (E-cadherin) but reduced mesenchymal markers (vimentin, CTGF and AXL). Furthermore, knockdown of TAZ in PC9/GR suppressed cell migration and invasion, which were considered functional hallmarks of EMT. These observations may partially explain the benefit of TAZ knockdown on EGFR-targeted therapy.

We further found stable TAZ knockdown dramatically reduced the expression of the classical Hippo target CTGF, a gene that regulated EMT-like transition. We showed that TAZ activated CTGF transcription by binding to and activating CTGF promoter. Consistent with our findings, previous studies showed that CTGF caused EMT-like cell fate mediated by TAZ-TEAD complex [16, 30, 31] and CTGF expression could confer resistance to chemotherapeutic agents through ERK1/2-dependent Bcl-xL/cIAP1 up-regulation in mastocarcinoma [32] and AMPK-dependent NF-κB pathway in osteosarcoma [33]. Tyrosine kinase AXL, a novel EMT marker and potential therapeutic target for overcoming EGFR inhibitor resistance [34], was also confirmed as a transcriptional target of TAZ in current study. The synergistic interaction between EGFR and AXL signaling showed that AXL transactivation mediated by associated EGFR amplified the response of a subset of downstream elements, quantitatively shifting emphasis of the downstream network across multiple pathways [35]. Moreover, this transactivation appeared to result from physical clustering interactions, which are quantitatively restricted to certain RTKs depending on a combination of intrinsic “affinity” and expression [36]. Therefore, CTGF and AXL also contribute to the potential explanations for TAZ-related sensitivity of EGFR-TKI therapeutics.

## Conclusions

In conclusion, we have provided convincing evidence that TAZ is a novel gene mediating tumorigenesis and EMT correlated with gefitinib sensitivity of lung adenocarcinoma cells harboring EGFR T790M mutation. Further confirmation of our findings using clinical patient samples will greatly facilitate our efforts in the sensitizing treatment of EGFR-TKI-resistant lung cancers in the future.

## Methods

### Cell culture

Human bronchial epithelial cell line 16HBE and lung adenocarcinoma cell line A549 were purchased from the Cell Resource Center (Shanghai Institutes for Biological Sciences, China). PC9, gefitinib-resistant PC9/GR, H1975 and cisplatin-resistant A549DDP cell lines were kindly provided by Prof. Hongbing Shen (Nanjing Medical University, Nanjing, China). All cells were grown in DMEM medium containing 10% FBS, 2mM L-glutamine and 100U/ml penicillin-streptomycin and incubated at 37°C with 5% CO^2^ in a humidified incubator. To maintain drug resistance, PC9/GR and H1975 cells were grown in DMEM containing 1 mg/ml gefitinib (Pure Chemistry Scientific Inc.) and then in drug free DMEM two days before experiments.

### Plasmid and transfection

The target sequences that shTAZ1 and shTAZ2 aimed to were GCGATGAATCAGCCTCTGAAT and AGGTACTTCCTCAATCACA. PC9/GR and H1975 cells reaching 80% confluence were transfected with pGPU6-shTAZ in the presence of lipofectamine 2000 (Invitrogen). PC9/GR-shNC, PC9/GR-shTAZ1 and PC9/GR-shTAZ2 cells were generated from G418 selection as described previously. The plasmid of pEX2-TAZ was purchased from Origene (Rockville, MD, USA). pEX2-TAZ-S51A was constructed using the QuickChange Mutagenesis Kit (Stratagene) according to the manufacture's protocol. The DNA of CTGF [nucleotide (nt) position -250 to -1] and AXL (nt -1180 to -235) promoters were amplified by PCR from genomic DNA extracted from 16HBE cells and subsequently cloned into pGL3-basic luciferase reporter vector (Promega).

### Real-time PCR

Total RNA was extracted from cells with TRIzol reagent (Invitrogen, San Diego, CA) according to the manufacturer’s instructions. cDNA was synthesized with the PrimeScript RT reagent kit (TaKaRa, Dalian, China). Quantitative RT-PCR was carried out with a SYBR Premix Ex Taq kit (TaKaRa) on a 7500 real time PCR system (ABI) as follows: 95°C for 30 s, 40 cycles at 95°C for 5 s, and 60°C for 30 s. The primers used were as follows: TAZ forward 5’-AGTACCCTGAGCCAGCAGAA-3’, reverse 5’-GATTCTCTGAAGCCGCAGTT-3’; CTGF forward 5’- CCCTCGCGGCTTACCGACTGG-3’, reverse 5’-CACAGGTCTTGGAACAGGCGC-3’; AXL forward 5’-TTTCCTGAGTGAAGCGGTCT-3’, reverse 5’-CATCTGAGTGGGCAGGTACA-3’; b-actin forward 5’-TGACGTGGACATCCGCAAAG-3’, reverse 5’-CTGGAAGGTGGACAGCGAGG-3’.

### Immunofluorescence and western blot

Cells were fixed with 4% paraformaldehyde, permeabilized with 0.1% Triton X-100/PBS, blocked in 2% BSA/PBS and probed with primary antibody against TAZ (1:200, Cell Signaling Technology, Inc.), followed by incubation with fluorescein isothiocyanate-conjugated secondary antibodies (1:100, Sigma). Nuclei were stained with DAPI. All immunofluorescence was visualized by confocal microscopy (LSM 700), and images were processed using Volocity software (PerkinElmer Life Sciences). Images were quantitated using Image J software. For western blot analysis, 20 μg of whole-cell lysates and nuclear fractions were used. Nitrocellulose membranes were incubated with antibodies against TAZ, E-cadherin (1:200, Cell Signaling Technology, Inc.), Vimentin (1:200, abcam), CTGF (1:200, Proteintech Group, Inc.), AXL (1:200, abcam), tAKT, pAKT, tERK and pERK (1:100, Cell Signaling Technology, Inc.), GAPDH (1:5000, Santa Cruz Biotechnology) and histone H3 (1:2000, abcam) followed by horseradish peroxidase-linked secondary antibody. The target protein was visualized by chemiluminescence (Denville Scientific, Metuchen, NJ).

### Cell viability assays

IC50 and inhibition rate of tumor growth was determined by the MTT (3-[4,5-dimethylthia-zol-2-yl]-2,5-diphenyltetrazolium bromide) assay (Sigma). Cancer cells were seeded into 96-well plates, and treated with gefitinib at different concentrations. After incubation, the media was replaced with 50 μL of MTT reagent (2 mg/mL) followed by further incubation in incubator for 2 h. Then, the media were removed and dimethylsulfoxide (DMSO) (150 μL) was added to each well. The absorbance at 560 nm was measured with a spectrophotometer.

### Colony formation assay

Stable PC9/GR cells knocking down TAZ or the empty control were trypsinized and replated at 2 × 10^2^ per well in 6-well plates. After 14 days in culture in DMEM supplemented with 10% FBS, colonies were fixed with 3.7% methanol and stained with Giemsa. Colonies containing at least 50 cells were scored.

### Xenograft tumor model

Four- to 6-week-old female nude mice were inoculated subcutaneously in the left and right thigh with 5 × 10^6^ PC9/GR-shNC and PC9/GR-shTAZ2 cells suspended in 100 μL of PBS to test tumorigenesis. Daily oral doses of gefitinib (75 mg/kg) in captisol solution were administered to tumor-bearing mice. Tumor size was measured twice a week to follow the drug response in animal model studies.

### Luciferase assay

Triplicates of 5 × 10^4^ 16HBE and PC9/GR cells in a 12-well plate were transfected with CTGF-luc or AXL-luc alone or together with TAZ or TAZ-S51A, using Lipofectamine 2000 (Invitrogen). As an internal transfection control, 10 ng of Renilla luciferase vector was also cotransfected in each sample. Luciferase activity was measured 2 days after transfection, using the Dual Luciferase Reporter Assay System (Promega) and the Turner Biosystems 20/20 Luminometer.

### Migration and invasion assays

Wound-healing assay was assessed by measuring the movement of monolayer cells into a acellular area created by a sterile plastic tip. The wound closure was photographed 24 h later by microscopy. Transwell migration and matrigel invasion assays were conducted using transwell plates with 8 μm pore size membranes (Corning Inc.). After incubation for 24 h, cells remaining in the upper side of the filter were removed with cotton swabs. The cells attached on the lower surface were fixed and stained. Cells were counted with five high power fields per membrane and results were presented as the mean number of cells migrated per field per membrane.

### Statistical analysis

Statistical analysis was performed using the SPSS software package (SPSS Standard version 16.0, SPSS Inc). Data were shown as mean ± SEM. For in vitro and in vivo experiments, independent-samples t-test was used for assessing the significance of difference between the treatment and control groups. P value <0.05 was considered as statistically significant.

## Electronic supplementary material

Additional file 1: Table S1: TaqMan-minor groove binder (MGB) probes were used in a real-time PCR-based assay for the rapid and accurate detection of PC9, PC9/GR and H1975 EGFR mutants. Loss of mutant delE746-A750 EGFR gene was observed in PC9 cells. PC9/GR cells harbored delE746-A750 plus T790M mutations. H1975 cells harbored L858R and T790M mutations. The IC50 values of PC9, PC9/GR and H1975 cells to gefitinb were 0.16 ± 0.01 μmol/L, 1.27 ± 0.08 μmol/L and 5.35 ± 0.22 μmol/L by MTT, respectively. Data are shown as means± SEM. n= 3. Statistical analyses were carried out using Student’s t-test. (JPEG 462 KB)

## References

[1] Bell DW, Gore I, Okimoto RA, Godin-Heymann N, Sordella R, Mulloy R, Sharma SV, Brannigan BW, Mohapatra G, Settleman J, Haber DA (2005). Inherited susceptibility to lung cancer may be associated with the T790M drug resistance mutation in EGFR. Nat Genet.

[2] Yoshida T, Zhang G, Smith MA, Lopez AS, Bai Y, Li J, Fang B, Koomen J, Rawal B, Fisher KJ, Chen AY, Kitano M, Morita Y, Yamaguchi H, Shibata K, Okabe T, Okamoto I, Nakagawa K, Haura EB (2014). Tyrosine Phosphoproteomics Identifies Both Codrivers and Cotargeting Strategies for T790M-Related EGFR-TKI Resistance in Non-Small Cell Lung Cancer. Clin Cancer Res.

[3] Suda K, Onozato R, Yatabe Y, Mitsudomi T (2009). EGFR T790M mutation: a double role in lung cancer cell survival?. J Thorac Oncol.

[4] Kim Y, Ko J, Cui Z, Abolhoda A, Ahn JS, Ou SH, Ahn MJ, Park K (2012). The EGFR T790M mutation in acquired resistance to an irreversible second-generation EGFR inhibitor. Mol Cancer Ther.

[5] Mitani A, Nagase T, Fukuchi K, Aburatani H, Makita R, Kurihara H (2009). Transcriptional coactivator with PDZ-binding motif is essential for normal alveolarization in mice. Am J Respir Crit Care Med.

[6] Hong JH, Hwang ES, McManus MT, Amsterdam A, Tian Y, Kalmukova R, Mueller E, Benjamin T, Spiegelman BM, Sharp PA, Hopkins N, Yaffe MB (2005). TAZ, a transcriptional modulator of mesenchymal stem cell differentiation. Science.

[7] Zhao B, Tumaneng K, Guan KL (2011). The Hippo pathway in organ size control, tissue regeneration and stem cell self-renewal. Nat Cell Biol.

[8] Azzolin L, Zanconato F, Bresolin S, Forcato M, Basso G, Bicciato S, Cordenonsi M, Piccolo S (2012). Role of TAZ as mediator of Wnt signaling. Cell.

[9] Dupont S, Morsut L, Aragona M, Enzo E, Giulitti S, Cordenonsi M, Zanconato F, Le Digabel J, Forcato M, Bicciato S, Elvassore N, Piccolo S (2011). Role of YAP/TAZ in mechanotransduction. Nature.

[10] Zhou Z, Hao Y, Liu N, Raptis L, Tsao MS, Yang X (2011). TAZ is a novel oncogene in non-small cell lung cancer. Oncogene.

[11] Xie M, Zhang L, He CS, Hou JH, Lin SX, Hu ZH, Xu F, Zhao HY (2012). Prognostic significance of TAZ expression in resected non-small cell lung cancer. J Thorac Oncol.

[12] Noguchi S, Saito A, Horie M, Mikami Y, Suzuki HI, Morishita Y, Ohshima M, Abiko Y, Mattsson JS, König H, Lohr M, Edlund K, Botling J, Micke P, Nagase T (2014). An Integrative Analysis of the Tumorigenic Role of TAZ in Human Non-Small Cell Lung Cancer. Clin Cancer Res.

[13] Yang N, Morrison CD, Liu P, Miecznikowski J, Bshara W, Han S, Zhu Q, Omilian AR, Li X, Zhang J (2012). TAZ induces growth factor-independent proliferation through activation of EGFR ligand amphiregulin. Cell Cycle.

[14] Yun CH, Mengwasser KE, Toms AV, Woo MS, Greulich H, Wong KK, Meyerson M, Eck MJ (2008). The T790M mutation in EGFR kinase causes drug resistance by increasing the affinity for ATP. Proc Natl Acad Sci U S A.

[15] Lau AN, Curtis SJ, Fillmore CM, Rowbotham SP, Mohseni M, Wagner DE, Beede AM, Montoro DT, Sinkevicius KW, Walton ZE, Barrios J, Weiss DJ, Camargo FD, Wong KK, Kim CF (2014). Tumor-propagating cells and Yap/Taz activity contribute to lung tumor progression and metastasis. EMBO J.

[16] Lai D, Ho KC, Hao Y, Yang X (2011). Taxol resistance in breast cancer cells is mediated by the hippo pathway component TAZ and its downstream transcriptional targets Cyr61 and CTGF. Cancer Res.

[17] Ji D, Deeds SL, Weinstein EJ (2007). A screen of shRNAs targeting tumor suppressor genes to identify factors involved in A549 paclitaxel sensitivity. Oncol Rep.

[18] Yuan M, Tomlinson V, Lara R, Holliday D, Chelala C, Harada T, Gangeswaran R, Manson-Bishop C, Smith P, Danovi SA, Pardo O, Crook T, Mein CA, Lemoine NR, Jones LJ, Basu S (2008). Yes-associated protein (YAP) functions as a tumor suppressor in breast. Cell Death Differ.

[19] Hall CA, Wang R, Miao J, Oliva E, Shen X, Wheeler T, Hilsenbeck SG, Orsulic S, Goode S (2010). Hippo pathway effector Yap is an ovarian cancer oncogene. Cancer Res.

[20] Huang JM, Nagatomo I, Suzuki E, Mizuno T, Kumagai T, Berezov A, Zhang H, Karlan B, Greene MI, Wang Q (2013). YAP modifies cancer cell sensitivity to EGFR and survivin inhibitors and is negatively regulated by the non-receptor type protein tyrosine phosphatase 14. Oncogene.

[21] Touil Y, Igoudjil W, Corvaisier M, Dessein AF, Vandomme J, Monté D, Stechly L, Skrypek N, Langlois C, Grard G, Millet G, Leteurtre E, Dumont P, Truant S, Pruvot FR, Hebbar M, Fan F, Ellis LM, Formstecher P, Van Seuningen I, Gespach C, Polakowska R, Huet G (2014). Colon cancer cells escape 5FU chemotherapy-induced cell death by entering stemness and quiescence associated with the c-Yes/YAP axis. Clin Cancer Res.

[22] Bai N, Zhang C, Liang N, Zhang Z, Chang A, Yin J, Li Z, Luo N, Tan X, Luo N, Luo Y, Xiang R, Li X, Reisfeld RA, Stupack D, Lv D, Liu C (2013). Yes-associated protein (YAP) increases chemosensitivity of hepatocellular carcinoma cells by modulation of p53. Cancer Biol Ther.

[23] Huo X, Zhang Q, Liu AM, Tang C, Gong Y, Bian J, Luk JM, Xu Z, Chen J (2013). Overexpression of Yes-associated protein confers doxorubicin resistance in hepatocellullar carcinoma. Oncol Rep.

[24] Mao B, Hu F, Cheng J, Wang P, Xu M, Yuan F, Meng S, Wang Y, Yuan Z, Bi W (2014). SIRT1 regulates YAP2-mediated cell proliferation and chemoresistance in hepatocellular carcinoma. Oncogene.

[25] Zhao Y, Khanal P, Savage P, She YM, Cyr TD, Yang X (2014). YAP-induced resistance of cancer cells to antitubulin drugs is modulated by a hippo-independent pathway. Cancer Res.

[26] Lai D, Visser-Grieve S, Yang X (2012). Tumour suppressor genes in chemotherapeutic drug response. Biosci Rep.

[27] Chung JH, Rho JK, Xu X, Lee JS, Yoon HI, Lee CT, Choi YJ, Kim HR, Kim CH, Lee JC (2011). Clinical and molecular evidences of epithelial to mesenchymal transition in acquired resistance to EGFR-TKIs. Lung Cancer.

[28] Lei QY, Zhang H, Zhao B, Zha ZY, Bai F, Pei XH, Zhao S, Xiong Y, Guan KL (2008). TAZ promotes cell proliferation and epithelial-mesenchymal transition and is inhibited by the hippo pathway. Mol Cell Biol.

[29] Cordenonsi M, Zanconato F, Azzolin L, Forcato M, Rosato A, Frasson C, Inui M, Montagner M, Parenti AR, Poletti A, Daidone MG, Dupont S, Basso G, Bicciato S, Piccolo S (2011). The Hippo transducer TAZ confers cancer stem cell-related traits on breast cancer cells. Cell.

[30] Zhang H, Liu CY, Zha ZY, Zhao B, Yao J, Zhao S, Xiong Y, Lei QY, Guan KL (2009). TEAD transcription factors mediate the function of TAZ in cell growth and epithelial-mesenchymal transition. J Biol Chem.

[31] Pobbati AV, Hong W (2013). Emerging roles of TEAD transcription factors and its coactivators in cancers. Cancer Biol Ther.

[32] Wang MY, Chen PS, Prakash E, Hsu HC, Huang HY, Lin MT, Chang KJ, Kuo ML (2009). Connective tissue growth factor confers drug resistance in breast cancer through concomitant up-regulation of Bcl-xL and cIAP1. Cancer Res.

[33] Tsai HC, Huang CY, Su HL, Tang CH (2014). CTGF increases drug resistance to paclitaxel by upregulating survivin expression in human osteosarcoma cells. Biochim Biophys Acta.

[34] Byers LA, Diao L, Wang J, Saintigny P, Girard L, Peyton M, Shen L, Fan Y, Giri U, Tumula PK, Nilsson MB, Gudikote J, Tran H, Cardnell RJ, Bearss DJ, Warner SL, Foulks JM, Kanner SB, Gandhi V, Krett N, Rosen ST, Kim ES, Herbst RS, Blumenschein GR, Lee JJ, Lippman SM, Ang KK, Mills GB, Hong WK, Weinstein JN, Wistuba II, Coombes KR, Minna JD, Heymach JV (2013). An epithelial-mesenchymal transition gene signature predicts resistance to EGFR and PI3K inhibitors and identifies Axl as a therapeutic target for overcoming EGFR inhibitor resistance. Clin Cancer Res.

[35] Zhang Z, Lee JC, Lin L, Olivas V, Au V, LaFramboise T, Abdel-Rahman M, Wang X, Levine AD, Rho JK, Choi YJ, Choi CM, Kim SW, Jang SJ, Park YS, Kim WS, Lee DH, Lee JS, Miller VA, Arcila M, Ladanyi M, Moonsamy P, Sawyers C, Boggon TJ, Ma PC, Costa C, Taron M, Rosell R, Halmos B, Bivona TG (2012). Activation of the AXL kinase causes resistance to EGFR-targeted therapy in lung cancer. Nat Genet.

[36] Meyer AS, Miller MA, Gertler FB, Lauffenburger DA (2013). The receptor AXL diversifies EGFR signaling and limits the response to EGFR-targeted inhibitors in triple-negative breast cancer cells. Sci Signal.

