# Supplementary figures and images for: Up-regulation of the Hippo pathway effector TAZ renders lung adenocarcinoma cells harboring EGFR-T790M mutation resistant to gefitinib
Source: Cell Biosci. 2015 Feb 5;5:7. doi: 10.1186/2045-3701-5-7 (PMC4429831; doi:10.1186/2045-3701-5-7)

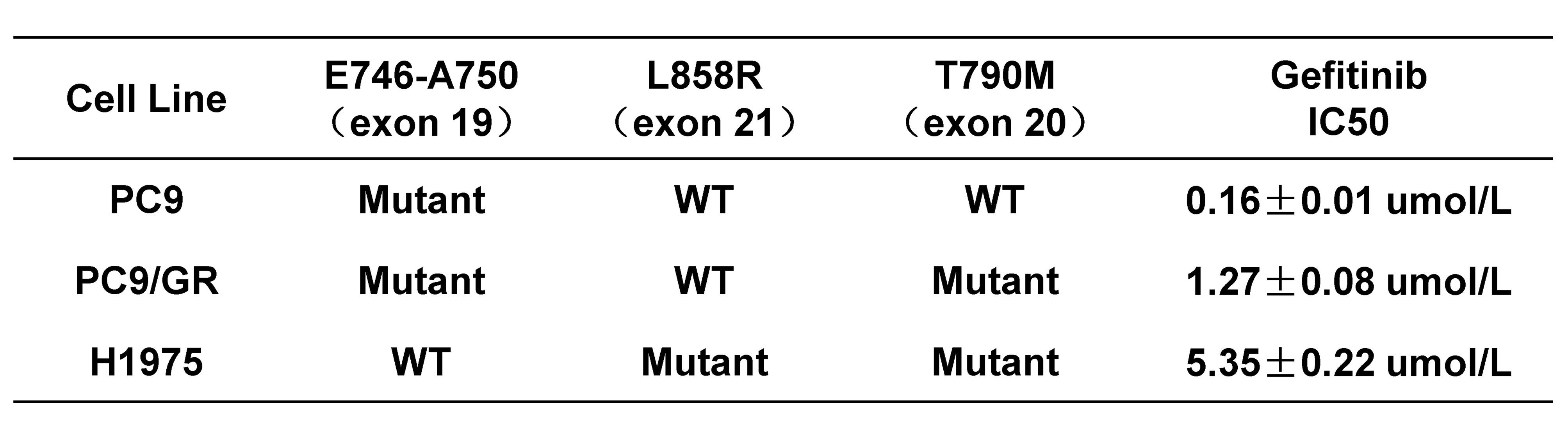

Supplement: Supplementary file 1 — Additional file 1: Table S1: TaqMan-minor groove binder (MGB) probes were used in a real-time PCR-based assay for the rapid and accurate detection of PC9, PC9/GR and H1975 EGFR mutants. Loss of mutant delE746-A750 EGFR gene was observed in PC9 cells. PC9/GR cells harbored delE746-A750 plus T790M mutations. H1975 cells harbored L858R and T790M mutations. The IC50 values of PC9, PC9/GR and H1975 cells to gefitinb were 0.16 ± 0.01 μmol/L, 1.27 ± 0.08 μmol/L and 5.35 ± 0.22 μmol/L by MTT, respectively. Data are shown as means± SEM. n= 3. Statistical analyses were carried out using Student’s t-test. (JPEG 462 KB) [file 13578_2014_217_MOESM1_ESM.jpeg]
